# Drinking Water Contaminants, Gene Polymorphisms, and Fetal Growth

**DOI:** 10.1289/ehp.7003

**Published:** 2004-05-26

**Authors:** Claire Infante-Rivard

**Affiliations:** Department of Epidemiology, Biostatistics and Occupational Health, Faculty of Medicine, McGill University, Montréal, Québec, Canada

**Keywords:** *CYP2E1* gene, disinfection by-products, drinking water, gene polymorphism, gene–environment interaction, intrauterine growth restriction, low birth weight, *MTHFR* gene, trihalomethanes

## Abstract

There are still many uncertainties regarding the risk of adverse pregnancy outcomes associated with exposure to drinking water disinfection by-products. In Montréal, Québec, Canada, we carried out a hospital-based case–control study including 493 cases of intrauterine growth restriction defined as birth weight below the 10th percentile for gestational age and sex, according to Canadian standards. Controls were babies (*n* = 472) delivered at the same hospital whose birth weight was at or above the 10th percentile, matched for gestational age, race, and sex. Exposure to total and specific trihalomethanes was measured using regulatory data collected by municipalities and the provincial Ministry of Environment. Residential history, water drinking, and shower habits during pregnancy, as well as known risk factors for intrauterine growth restriction, were measured with a face-to-face interview with all mothers. Mothers and newborns were characterized for two genetic polymorphisms, one in the *CYP2E1* gene (G1259C), and another in the 5,10-methylenetetrahydrofolate reductase (*MTHFR*) gene (C677T). Exposure to specific and total trihalomethanes from drinking water, determined for 458 cases and 426 controls, did not result in an increased risk of intrauterine growth restriction. However, significant effect modification was observed between newborns with and without the *CYP2E1* variant; among newborns with the variant, the adjusted odds ratio for intrauterine growth restriction associated with exposure to average total trihalomethanes above the 90th percentile (corresponding to 29.4 μg/L) was 13.20 (95% confidence interval, 1.19–146.72). These findings suggest that exposure to trihalomethanes at the highest levels can affect fetal growth but only in genetically susceptible newborns.

Chlorination by-products in drinking water come from the reaction of chlorine with organic material in the water. This reaction occurs naturally or originates from municipal, agricultural, and industrial wastes. Trihalomethanes (THMs) such as chloroform, bromoform, bromodichloromethane (BDCM), and chlorodibromomethane are the most prevalent class of disinfection by-products (DBPs) found in treated water. From the toxicologic literature, chloroform appears to affect fetal development (Geveker Graves et al. 2001), by mechanisms that have not yet been elucidated. In the last decade, a number of epidemiologic studies have been carried out to determine the effect of DBPs on adverse pregnancy outcomes (Bove et al. 2002). Two recent reviews propose that the weight of the evidence, although moderate and not fully conclusive, is in favor of an association between DBPs and fetal growth restriction (Bove et al. 2002; Geveker Graves et al. 2001). Most of the previous studies were based on information from birth records, and despite the fact that the number of records included was usually large, the information that was available on other risk factors for fetal growth, or on other personal variables influencing exposure to DBPs, was often limited.

The primary enzyme involved in the metabolism of low doses of chloroform is CYP2E1 (Meek et al. 2002). My group has previously shown that a polymorphism in the *CYP2E1* gene can modify the effect of water contaminants (Infante-Rivard et al. 2002a). Another enzyme, 5,10-methylenetetrahydro-folate reductase (MTHFR), together with folic acid, is involved in the remethylation of homocysteine to methionine, as well as in the methylation of DNA, proteins, and phospholipids (Botto and Yang 2000). Alston (1991) reported that vitamin B_12_-dependent methionine biosynthesis could be inhibited by chloroform. Common polymorphisms in the *MTHFR* gene have been identified (Botto and Yang 2000). To my knowledge, no study has considered the role of genetic polymorphisms on the relationship between DBPs and fetal growth.

My group carried out a study on genetic and metabolic risk factors for intrauterine growth restriction (IUGR) (Infante-Rivard et al. 2002b, 2003a, 2003b). In the course of the study, we also collected personal and environmental information to analyze the association between chemical water contaminants and fetal growth.

## Materials and Methods

### Study subjects.

Details on study subjects have been reported elsewhere (Infante-Rivard et al. 2002b). Briefly, cases were newborns whose birth weight was below the 10th percentile for gestational age and sex, based on Canadian standards (Arbuckle et al. 1993). All cases seen at the largest university-based mother–child center in Montréal between May 1998 and June 2000 who were born singleton, alive after the 24th week of gestation, and without severe congenital anomalies were eligible for the study. During that period, 505 newborns met the eligibility criteria, and 493 were included in the study (97.6%). Controls were born at the same hospital and met the same eligibility criteria, except that their birth weight was at or above the 10th percentile. They were matched to cases for gestational week, sex, and race (white, black, Hispanic/Amerindian, and Asian) and usually born within 1 week of the matched case subject. Of those identified, 480 controls were invited to participate, and 472 accepted (98.3%). The project was approved by the hospital ethics committee. An informed consent was signed by the mother to collect cord and maternal blood.

### Interview.

A face-to-face interview with all mothers of cases and controls was carried out in French or English at the hospital, generally within 2 days of delivery. It included questions about demographic factors, complications of pregnancy, maternal chronic diseases, obstetric history, and smoking. The medical record was used for variables such as height and weight and to confirm pregnancy diseases. To determine exposure to water contaminants for each pregnancy trimester until delivery, we collected the following information: maternal residential history, source of drinking water (community, private well, bottled), use and type of domestic water filter, average number of glasses of water per day at home or elsewhere (including those with reconstituted frozen fruit juices), usual way of consuming tap water (directly from tap, after refrigeration), average number of showers per week, and usual duration of showers.

### Exposure ascertainment.

For the study period, exposure to THMs from drinking water according to place of residence was obtained from regulatory data collected by municipalities and the Ministry of Environment. There were 189 distribution systems involved; although for most systems there were multiple measurements on the same date, I was only provided with average measures. Of the 965 women in the study, 10 lived in other Canadian provinces, 37 lived in other countries, and there was no address for 2 others, leaving 916 study women reporting addresses in the province. Overall, THM information was available for 884 (91.6%) of the study women (458 cases and 426 controls).

### Exposure from drinking water.

Estimates of exposure levels to total and specific THMs from drinking water were tabulated first as average level at the tap (from treatment plant data) over the pregnancy period [(sum of concentration *i* × duration in days at level *i* based on residence) ÷ (total number of pregnancy days)]; this measure was then categorized at the 90th percentile of the distribution for cases and controls. Another index was the cumulative level over the pregnancy period (sum of concentration *i* × duration in days at level *i*); it was also categorized at the 90th percentile of the distribution. When the source of drinking water was exclusively well water or bottled water, the exposure levels for drinking water were set at zero. Finally, the estimated average level of THMs at the tap from the municipal distribution system was multiplied by the number of glasses of tap water per day averaged over pregnancy. Another version of this index included applying an arbitrary weight of 0.9 to the average number of tap water glasses if a filter was used or if the water was refrigerated before consumption.

### Exposure from showering.

Exposure to THMs from showering was set to zero for residences using exclusively well water, others were assigned their network level. An index of exposure to THMs from showering was defined as frequency of showers per week (times duration) multiplied by the average levels of exposure to THMs at the tap from the distributing network.

### Genotyping.

Polymerase chain reaction (PCR) allele-specific oligonucleotide hybridization assays have been used to genotype the polymorphism G1259C (a G-to-C substitution at position 1259 in the promoter) that defines the allele CYP2E1*5 (Infante-Rivard et al. 2002a) as well as the *MTHFR* C677T polymorphism (Infante-Rivard et al. 2002b).

### Statistical analysis.

Out of 493 cases and 472 controls, 451 were matched for gestational week, sex, and race. Because the matching involved only categorical factors, odds ratios (ORs) and 95% confidence intervals (CIs) were estimated using unconditional logistic regression analysis, allowing all study subjects to be included. I included race, sex, and gestational age as confounding variables in all analyses, as well as the following risk factors known to be associated with IUGR: weight gain during pregnancy, prepregnancy body mass index (BMI), parity, history of preeclampsia, prior history of IUGR, primiparity, and smoking during pregnancy. I also tested for gene–environment interactions, that is, whether the effect of water contaminants (total THMs and chloroform in tap water) was modified by newborn and maternal genetic variants (one or two variant alleles vs. none), using a heterogeneity chi-square test (Hills and De Stavola 2002).

## Results

Table 1 provides some background maternal characteristics for cases and controls. As expected, case mothers had gained less weight during pregnancy and had a lower BMI before pregnancy. In addition, they were more likely to be older, to have smoked during the third trimester of pregnancy, to be primiparous, to have preeclampsia, and to report a previous pregnancy with IUGR.

Table 2 shows the distribution of exposure variables such as different THMs as well as showering and drinking-water habits. The only notable difference between cases and controls was the use of domestic water filters, which was higher among controls.

Table 3 shows the results for exposure to specific and total THMs in drinking water using the 90th percentile cutoff for average level of exposure. No increased risk was observed for any of the specific THMs or for total THMs. All reported ORs were fully adjusted. Using a cutoff at the 95th percentile (instead of the 90th) for average level of exposure, I estimated an OR of 1.17 (95% CI, 0.60–2.29) for chloroform and of 1.26 (95% CI, 0.65–2.45) for total THMs. I also estimated ORs for cumulative exposure to specific and total THMs in drinking water and found that results were very similar to those for average exposure (data not shown). Using the other indices for drinking water and the index for showering, I observed no increased risks (data not shown).

Table 4 shows the adjusted ORs for exposure to average levels of chloroform and total THMs from drinking water (contrasting the group above the 90th percentile with the group at or below the 90th), according to whether the newborn or the mother carried one or two variant alleles, as opposed to none. The risk of IUGR associated with exposure to total THMs was different between newborn carriers and noncarriers of the *CYP2E1* variant. An increased risk was also observed among the newborn carriers for exposure to chloroform, as well as among mother carriers for both exposures (chloroform and total THMs), but the risks were not statistically different across the gene strata. I also observed statistical heterogeneity in the risk of IUGR between newborns carriers and noncarriers of the *CYP2E1* variant for exposure to average levels of chloroform and total THMs measured as numerical variables (data not shown). No effect modification was observed when contrasting carriers and noncarriers of the *T* allele in the *MTHFR* C677T gene. No significant effect modification was observed between newborn or maternal carriers and noncarriers of either polymorphism when exposure was defined with the other indices for drinking water or for showering.

## Discussion

My results for the association between exposure to water contaminants were largely negative, whether average or cumulative levels from drinking water at the tap were used or when I also accounted for drinking water and showering habits. There were some indications that, with increased genetic susceptibility, especially in the newborn, measured by the presence of a variant in the *CYP2E1* gene, exposure to total THMs was associated with substantial risk. I know of no similar results.

The results from previous studies are mixed with respect to IUGR (often referred to as small for gestational age): Four studies reported associations (Bove et al. 1995; Gallagher et al. 1998; Kramer et al. 1992; Wright et al. 2003), and five did not (Dodds et al. 1999; Jaakkola et al. 2001; Kallen and Robert 2000; Savitz et al. 1995; Yang et al. 2000). In the present study, selection bias was unlikely. Exposure assessment at the personal level was more detailed than in most previous studies because I accounted for the use of bottled water as drinking water, the drinking habits, and the showering habits (although not the bathing habits). A substantial proportion of women were drinking only bottled water, which influenced the levels of exposure to THMs reported in the study. Despite these positive features, misclassification of exposure to water contaminants was most certainly present; in particular, better measures could be achieved if assigned levels were based on specific locations within the distribution systems when multiple locations within the system were sampled. Another advantage of this study, compared with many of the previous studies using birth records, was the extensive control for confounding; gestational age, child’s sex, race, maternal smoking, primiparity, weight gain during pregnancy, BMI, previous IUGR, and pregnancy hypertension are all known risk factors for IUGR for which I was able to adjust.

Despite the absence of association between exposure to THMs and IUGR, the adverse effects of exposure of THMs were uncovered when taking into account genetic susceptibility. The study included subjects from many racial backgrounds; confounding by ethnicity, known as population stratification, can bias the results of case–control studies with genetic risk factors. However, I adjusted for race in all analyses. Other confounders for IUGR were measured and controlled for in the analysis.

The mechanism by which exposure to THMs affects fetal growth is not known; among humans, almost all studies have been epidemiologic, and therefore other types of studies addressing mechanisms are not available. A recent toxicologic study hypothesized that BDCM could disrupt the synthesis and/or secretion of placental syncytiotrophoblast-derived chorionic gonadotropin (Chen et al. 2003). The authors tested whether BDCM targets trophoblasts by examining the effect of BDCM on chorionic gonadotropin secretion by primary cultures of human trophoblasts. The results showed that BDCM reduced the secretion of immunoreactive and bioactive chorionic gonadotropin, and thus the component appears to target human placental trophoblasts. Trophoblasts are the sole source of chorionic gonadotropin during normal human pregnancy; thus, a decrease in the amount of this bioactive hormone could have adverse effects on pregnancy outcome. However, much more work is still needed to elucidate the possible effects on human fetal growth.

A few years ago, Chen et al. (1996) were the first to suggest that carriers of the common *MTHFR* C677T polymorphism could be at higher risk for the effects of chloroform in drinking water. MTHFR is involved in the metabolism of methionine and homocysteine through a mechanism that is vitamin B_12_ dependent; as suggested by Alston (1991), the latter could be inhibited by chloroform. Among carriers of the *T* allele, in particular the homozygotes, the transformation of homocysteine to methionine is less efficient and possibly the exposure to chloroform could inhibit this transformation even more. In this study, I found no indication that *MTHFR* C677T will modify the effect of exposure to chloroform. It is likely that this is not a promising hypothesis after all, at least for fetal growth.

The tested variant in *CYP2E1* is in the regulatory region and is associated with an increased transcriptional activity (Hayashi et al. 1991). The carriers would be expected to have an increased metabolism of THMs resulting in the production of activated metabolites. Our results are coherent with this hypothesis. This indication for gene–environment interaction should lead to more similar investigations because it is very unlikely that only one polymorphic gene is involved in the metabolism of THMs.

In conclusion, in the present study I was unable to show effects of exposure to THMs from DBPs on the risk of IUGR. However, among newborn carriers of a *CYP2E1* gene variant, important effects were observed. These results will need confirmation. They also suggest that accounting for genetic susceptibility is a sensible way to study the effects of environmental exposures when there is information on the candidate genes involved in the metabolism of these agents.

## Figures and Tables

**Table 1:** Distribution of maternal characteristics between cases (*n* = 493) and controls (*n* = 472).

Characteristic	Cases	Controls
Race [no. (%)]		
White	330 (66.9)	333 (70.5)
Black	117 (23.7)	110 (23.3)
Asian	24 (4.8)	13 (2.7)
Hispanic/Amerindian	22 (4.4)	16 (3.4)
Age ≥ 36 years [no. (%)]	86 (17.4)	70 (14.8)
Schooling ≤ 12 years [no. (%)]	108 (21.9)	96 (20.3)
Prepregnancy BMI (mean ± SD)	22.8 ± 4.3	23.2 ± 5.2
Weight gain during pregnancy, kg (mean ± SD)	12.7 ± 5.5	14.4 ± 5.6
Primiparous [no. (%)]	321 (65.2)	234 (49.6)
Preeclampsia [no. (%)]	69 (14.0)	12 (2.5)
Previous IUGR among parous [no. (%)]	66 (38.4)	23 (9.7)
Any cigarette smoking in 3rd trimester [no. (%)]	112 (22.7)	72 (15.7)

**Table 2:** Distribution of exposure variables between cases and controls.

Variable	Cases	Controls
THM concentration (μg/L) at the tap (mean ± SD)		
Chloroform	11.84 ± 18.19	11.58 ± 16.31
Bromoform	0.42 ± 0.62	0.36 ± 0.65
BDCM	4.34 ± 2.94	4.24 ± 3.42
Chlorodibromomethane	2.21 ± 1.95	2.08 ± 2.30
Total THM	18.74 ± 19.76	18.26 ± 18.89
Other exposure characteristics		
Drinking bottled water (%)	21.9	26.4
Private well (%)	0.66	0.95
Use of domestic water filter at tap (%)	14.7	9.9
Glasses of water/day*^a^* (mean ± SD)	6.8 ± 4.7	6.7 ± 4.2
No. of weekly showers (mean ± SD)	6.8 ± 3.9	6.9 ± 3.8
Duration of showers, min (mean ± SD)	12.6 ± 8.3	13.1 ± 7.3

a Includes water mixed with frozen juices.

**Table 3:** Adjusted*^a^* ORs (95% CIs) for IUGR in relation to exposure to specific and total THMs in drinking water measured as average levels at the tap.

Exposure index	Value at cutoff (μg/L)	OR (95% CI)
Average level ( > 90th percentile vs. ≤ 90th percentile)		
Chloroform	23.7	1.06 (0.63–1.79)
Bromoform	1.22	2.44 (0.19–31.10)
BDCM	6.3	0.84 (0.50–1.43)
Chlorodibromomethane	3.9	0.62 (0.27–1.44)
Total THM	29.4	0.97 (0.57–1.62)

a Adjusted for gestational age, sex, race, mother’s weight gain during pregnancy, prepregnancy BMI, smoking during the third trimester, primiparity, preeclampsia in the current pregnancy, and previous IUGR.

**Table 4:** Adjusted*^a^* ORs (95% CIs) for exposure to THMs (chloroform and total THMs) in drinking water measured as average level at the tap, according to newborn and maternal polymorphisms in the *CYP2E1* and *MTHFR* genes.

			OR (95% CI)*^b^*	
Gene	Cases (*n*)	Controls (*n*)	Chloroform	Total THMs
Newborns				
CYP2E1*5 (G1259C)				
Wild type	385	375	0.99 (0.57–1.74)	0.82 (0.47–1.45)
1 or 2 variant alleles	45	37	5.62 (0.82–38.39)	13.20 (1.19–146.72)*
*MTHFR* C677T				
Wild type	239	212	1.78 (0.82–3.87)	1.63 (0.72–3.71)
1 or 2 variant alleles	195	204	0.83 (0.38–1.54)	0.76 (0.38–1.54)
Mothers				
CYP2E1*5 G1259C				
Wild type	395	380	0.88 (0.50–1.54)	0.83 (0.48–1.44)
1 or 2 variant alleles	57	39	4.40 (0.73–26.42)	6.54 (0.59–71.45)
*MTHFR* C677T				
Wild type	244	214	1.00 (0.46–2.18)	0.98 (0.46–2.10)
1 or 2 variant alleles	212	206	1.12 (0.56–2.32)	0.94 (0.47–1.89)

a Adjusted for gestational age, sex, race, mother’s weight gain during pregnancy, prepregnancy BMI, smoking during the third trimester, primiparity, preeclampsia in the current pregnancy, and previous IUGR.

b For exposure defined as average level (> 90th percentile vs. ≤ 90th percentile).

* Chi-square (1 degree of freedom) for effect modification = 4.87; *p* = 0.027.
